# Editorial: New Directions in the Management of Status Epilepticus

**DOI:** 10.3389/fneur.2018.00994

**Published:** 2018-11-23

**Authors:** Batool F. Kirmani, Ashok K. Shetty, Lee A. Shapiro

**Affiliations:** ^1^Epilepsy Center, Centra Neurosciences, CMG Neurology Center, Lynchburg, VA, United States; ^2^Institute for Regenerative Medicine, Department of Molecular and Cellular Medicine, Texas A&M University College of Medicine, College Station, TX, United States; ^3^Department of Neuroscience and Experimental Therapeutics, Texas A&M University College of Medicine, Bryan, TX, United States

**Keywords:** epilepsy, stautus epilepticus, seizures, anticonvulsants, autoimmune epilepsy

Status Epilepticus (SE) is a neurological emergency and has high morbidity and mortality. The International League Against Epilepsy (ILAE) recently updated their definition to specify that, “SE is a condition resulting either from the failure of the mechanisms responsible for seizure termination or from the initiation of mechanisms, which lead to abnormally, prolonged seizures.” Such phenomena can lead to long-term neurological complications due to neuronal death, glia, neurological injury, aberrant neuroplasticity, oxidative stress and inflammation, and alteration of neuronal networks. Depending upon the type and duration of SE, these mechanisms are quite variable. Therefore, in response to the updated definition of SE, novel avenues of research are required to address the specified involvement of the underlying mechanisms and pathophysiology resulting in the development of and outcomes from SE.

Improving the basic science understanding of SE will facilitate essential clinical trials. One can envision such experiments to include device and compound-based technological interventions directed at aborting the seizure activity and improving clinical outcomes. Benzodiazepines remain one of the cornerstones of treatment, and studies are underway to study new delivery options, including intranasal, buccal, and intramuscular midazolam, in addition to rectal diazepam, with the goal of aborting the seizure activity outside the hospitals, as rapidly as possible. Approved and off-label anticonvulsants, such as phenytoin, phenobarbital, valproate, topiramate, levetiracetam, lacosamide, steroids, immunosuppressants, and neuroprotective compounds, have also shown some efficacy at treating SE. However, substantial challenges remain in optimally managing SE and minimizing the short- and long-term complications. Such difficulties can be overcome by innovative approaches targeting the underlying mechanisms of neuronal excitability, glia, neuronal death, neuroplasticity, oxidative stress, inflammation, and neuroinflammation.

The book comprises six original research articles and four reviews. Collectively, the materials provide insights into the pathophysiology, clinical presentation, treatment, recent advances and future directions in the management of SE, with the goal of providing an in-depth view and advancing the field to improve management of SE.

The book opens with an original research article by Kristin Phillips et al. which showed the role of hypothermia as a neuroprotective agent for preventing the development of calcium plateau against SE-induced delayed hippocampal injury. Hypothermia-mediated neuroprotection after pilocarpine-induced SE was evident from decreased Fluoro-Jade C+ neurons in the hippocampus. The second original article by Matos et al. described SE-induced changes in spontaneous locomotor activity and the temporal expression of genes related to circadian rhythms (Clock, Bmal1, Cry1, Cry2, Per1, Per2, and Per3) in the hippocampus at both early post-SE and chronic epilepsy phases. Authors propose that seizures can act as a non-photic cue and altered temporal expression of clock genes likely contributes to the pathogenesis of mesial temporal lobe epilepsy. The third original article by Hutson et al. presented an interesting case study which showed evidence of brain dynamics resetting after successful anticonvulsant treatment following SE utilizing stereo encephalography (SEEG) data.

A review by Kirmani et al. conferred the current literature about autoimmune SE including therapeutic options and future directions. An original research article by Wyatt-Johnson et al. reported that SE-induced morphological alterations in microglia at different time-points and discussed the role of such changes on epileptogenesis. Another research article by Kortland et al. addressed the socioeconomic outcome and quality of life outcome in adults after status epilepticus in their original article. The authors conducted a multicenter, longitudinal, matched case-control analysis and concluded that relatively favorable outcomes seen in patients with refractory and super refractory SE as compared to non-refractory SE cases underlying the need of effective therapeutic choices.

An original research article by Bertoglio et al. compared the effects of two different protocols of kainate-induced SE in two strains of rats on neurodegeneration and chronic epilepsy development. The findings revealed that severe neuron loss after SE does not necessarily correlate with a higher seizure rate in the chronic phase after SE. In a review article, Castro et al. discussed the efficacy and promise of resveratrol, a phytoalexin found in the skin of red grapes, for easing SE-induced neurodegeneration, neuroinflammation, aberrant neurogenesis and for restraining the evolution of SE-induced brain injury into a chronic epileptic state. Sharma et al. by reviewing methods of induction and characterization of behavioral SE and EEG correlates in mice and rats, highlighted the advantages of a repeated low dose of kainate protocol for minimizing the variability in the initial SE severity and reducing the mortality rate. The last original article by Lucchi et al. described the role peroxisome proliferator-activated receptor gamma in the anticonvulsant properties of EP-80317, a Ghrelin receptor antagonist in pilocarpine-induced SE rat model and repeated 6 Hz corneal stimulation model in mice.

## Author contributions

All authors listed have made a substantial, direct and intellectual contribution to the work, and approved it for publication.

### Conflict of interest statement

The authors declare that the research was conducted in the absence of any commercial or financial relationships that could be construed as a potential conflict of interest.

